# Rotor Tracking Using Phase of Electrograms Recorded During Atrial Fibrillation

**DOI:** 10.1007/s10439-016-1766-4

**Published:** 2016-12-05

**Authors:** Caroline H. Roney, Chris D. Cantwell, Norman A. Qureshi, Rasheda A. Chowdhury, Emmanuel Dupont, Phang Boon Lim, Edward J. Vigmond, Jennifer H. Tweedy, Fu Siong Ng, Nicholas S. Peters

**Affiliations:** 1 Department of Bioengineering, Imperial College London, South Kensington Campus, London, SW7 2AZ UK; 2 IHU Liryc, Electrophysiology and Heart Modeling Institute, fondation Bordeaux Université, F-33600 Pessac-Bordeaux, France; 3 Department of Aeronautics, Imperial College London, South Kensington Campus, London, SW7 2AZ UK; 4 National Heart and Lung Institute, Imperial College London, 4th floor Imperial Centre for Translational and Experimental Medicine, Hammersmith Campus, Du Cane Road, London, W12 0NN UK

**Keywords:** Cardiac arrhythmia, Phase singularity mapping, Electrogram analysis

## Abstract

Extracellular electrograms recorded during atrial fibrillation (AF) are challenging to interpret due to the inherent beat-to-beat variability in amplitude and duration. Phase mapping represents these voltage signals in terms of relative position within the cycle, and has been widely applied to action potential and unipolar electrogram data of myocardial fibrillation. To date, however, it has not been applied to bipolar recordings, which are commonly acquired clinically. The purpose of this study is to present a novel algorithm for calculating phase from both unipolar and bipolar electrograms recorded during AF. A sequence of signal filters and processing steps are used to calculate phase from simulated, experimental, and clinical, unipolar and bipolar electrograms. The algorithm is validated against action potential phase using simulated data (trajectory centre error <0.8 mm); between experimental multi-electrode array unipolar and bipolar phase; and for wavefront identification in clinical atrial tachycardia. For clinical AF, similar rotational content (*R* ^2^ = 0.79) and propagation maps (median correlation 0.73) were measured using either unipolar or bipolar recordings. The algorithm is robust, uses standard signal processing techniques, and accurately quantifies AF wavefronts and sources. Identifying critical sources, such as rotors, in AF, may allow for more accurate targeting of ablation therapy and improved patient outcomes.

**Electronic supplementary material:**

The online version of this article (doi:10.1007/s10439-016-1766-4) contains supplementary material, which is available to authorized users.

## Introduction

Electrogram signals measured during cardiac fibrillation are inherently complex, with multi-component deflections and often limited temporal organisation. Phase mapping transforms them from direct measurements of voltage over time to signals that capture the wavefront dynamics through the activation-recovery cycle of the underlying tissue by elucidating periodicity not immediately apparent in the raw signal. One advantage of phase maps derived from spatially distributed electrogram signals is that centres of rotational activity manifest clearly as phase singularities.^16^ Identifying these phase singularities can be used to locate the cores of spiral waves, which are a proposed mechanism underlying cardiac fibrillation.

During clinical ablation procedures, voltage data are recorded in the form of unipolar or bipolar electrograms. Analysis and interpretation of these recordings are used to inform ablation strategies. Recent ablation approaches using a non-invasive mapping technology, which reconstructs unipolar electrograms from body surface unipolar electrograms, target areas of high phase singularity density, since these indicate electrical driver locations.^17^ Bipolar electrograms are often preferred clinically since they implicitly eliminate far-field electrical contributions. Unfortunately, AF bipolar electograms are often multicomponent fractionated signals with a varying cycle length and complex activation patterns, leading to difficulties in assigning activation times and windows with which to construct isochronal maps^6^ and challenges in calculating phase appropriately. Current techniques used to analyse fibrillatory bipolar electrogram data include frequency analysis such as dominant frequency or organisational index calculations^30^; peak-to-peak voltage calculations; fractionation scoring analysis^22^; and Shannon entropy analysis.^14^ However, these techniques typically assign a single summary statistic per electrode, which can be hard to interpret spatially due to the continuously evolving dynamics of fibrillatory activity; as such, these techniques show limited success in guiding ablation strategies.

Phase mapping has the potential to overcome the limitations of current techniques for analysing bipolar electrograms, because it provides spatiotemporal information and does not require the assignment of activation times or windows. Many optical mapping and simulation studies have used phase mapping techniques to analyse wavefront dynamics from action potential (AP) data.^10^ More recently phase mapping using unipolar electrograms has been applied to ventricular fibrillation (VF)^9^,^24^ and AF data.^18^,^20^ However, currently there are no established methodologies for calculating phase of bipolar electrograms because the accurate calculation of electrogram phase during AF is challenging due to the non-sinusoidal and fractionated nature of the electrograms.

A method for phase mapping of atrial unipolar electrograms using a sinusoidal recomposition technique was developed by Kuklik *et al*.^20^ The signal is first represented as a sum of sinusoidal wavelets of amplitude proportional to the negative slope of the electrogram and period equal to the mean cycle length. This sinusoidal reconstruction behaves well for phase mapping using the Hilbert Transform, and captures the timings of traditionally defined activation, because it is based on the size of the negative electrogram derivative. They showed that their method is superior to calculating the phase of the raw electrogram, but it has the disadvantage that it may not work well in the case of varying cycle length.

In this paper, we describe a robust methodology for calculating phase from either unipolar or bipolar electrogram data, significantly extending our previous work^28^ by generalising the algorithm and validating it extensively on real data. The unipolar and bipolar phase methodologies are validated using both simulated electrograms generated by a computer model of fibrillation, and using cell-culture electrogram recordings obtained using micro-electrode arrays. The techniques are then applied to clinically acquired multi-polar catheter data. Data recorded during atrial tachycardia are used for clinical validation of the algorithms since activation times are known with a degree of certainty in this condition. The rotational content, activation patterns and phase angle correlation are compared for unipolar and bipolar phase during atrial fibrillation.

To our knowledge this represents the first published methodology appropriate for calculating phase of both unipolar and bipolar electrograms from simulated, experimental or clinical modalities.

## Materials and Methods

### Data Acquisition

#### Simulated Data

Spiral wave simulations were performed on a two-dimensional left atrial surface. The surface triangulation was generated by segmentation of MRI data using ITK-snap,^34^ and the resulting mesh was cut at the mitral valve and four pulmonary veins using the mesh manipulation package Blender (http://www.blender.org). The surface was remeshed using Gmsh^15^ to produce a triangulation with elements of approximately equal characteristic length. The cardiac electrophysiology solver,^7^ built on the Nektar++ high-order spectral/hp element framework,^5^ was used for simulations. The Courtemanche ionic model,^11^ incorporating changes representing electrical remodelling in AF,^12^ was used to model the human atrial action potential and the monodomain formulation was used for action potential propagation. An S1-S2 pacing protocol was used to induce a pair of counter-rotating rotors.

Unipolar electrograms were calculated (sampling frequency 2 kHz) following Sato *et al.* 31 at electrode locations approximating nine catheters distributed throughout the chamber, each modelled as an Archimedean spiral of diameter 2 cm. Electrodes were projected 2 mm endocardially along the surface normal vector. For each catheter, bipolar electrograms were calculated as the difference between adjacent pairs of the twenty unipolar electrograms, consistent with clinical recordings.

AP data (sampling frequency 1 kHz) were post-processed to calculate AP phase by initially removing the mean from the signal using a pseudo empirical mode decomposition technique,^3^ and then taking the Hilbert transform of the zero-mean signal.

#### Microelectrode Array Data

Unipolar field potentials were recorded (10 s duration, 50 kHz sampling frequency) using micro-electrode arrays (MEAs) from a spontaneously fibrillating monolayer of HL-1 clone 6 cells (mouse atrial myocytes). The HL-1 cells were originally obtained from Dr W. C. Claycomb (Louisiana State University Health Centre, New Orleans, LA, USA) and then separated into stable sub-clones by Dr Emmanuel Dupont.^13^ Confluent cells were seeded over an area of 0.5 cm^2^ at the centre of a 1.4 × 1.4 mm micro-electrode array (Multichannel Systems GMBH, GMBH), consisting of an 8-by-8 grid, with inter-electrode spacing 200 *μ*m and an electrode diameter of 30 *μ*m. Bipolar electrograms were constructed as the difference between unipolar signals in the vertical direction for each pair of rows, to give a 7-by-8 grid of bipolar electrograms.

#### Clinical Data

All data were obtained with informed consent under ethical approval from the Health Research Authority Ref 13/LO1169. Electrograms and electrode locations were recorded during atrial arrhythmias from the left atrium of patients at the beginning of ablation procedures, using multipolar spiral AFocusII catheters and the Ensite Velocity electroanatomic mapping system (St Jude Medical, Inc). Catheters were positioned at multiple sites on the posterior wall and roof of the left atrium for 11 patients with AF (6–17 catheter recording locations per patient; 127 in total). Unipolar and bipolar electrograms were recorded at a sampling frequency of 2.0345 kHz from all electrodes simultaneously for between 16 and 106 s (mean 34 s). For validation of the phase algorithms, electrograms (mean duration 28 s) were also recorded from one patient with an atrial tachycardia (AT). For both AF and AT recordings, signals indicating poor contact with the tissue were removed from the analysis using a patient-specific low-voltage threshold, which was tuned through visual inspection of electrograms by an experienced clinician. To validate AT wavefront directions calculated from phase, an AT local activation time map constructed using the Ensite Velocity system was used as a reference.

### Unipolar and Bipolar Phase Calculation

**Figure 1:**
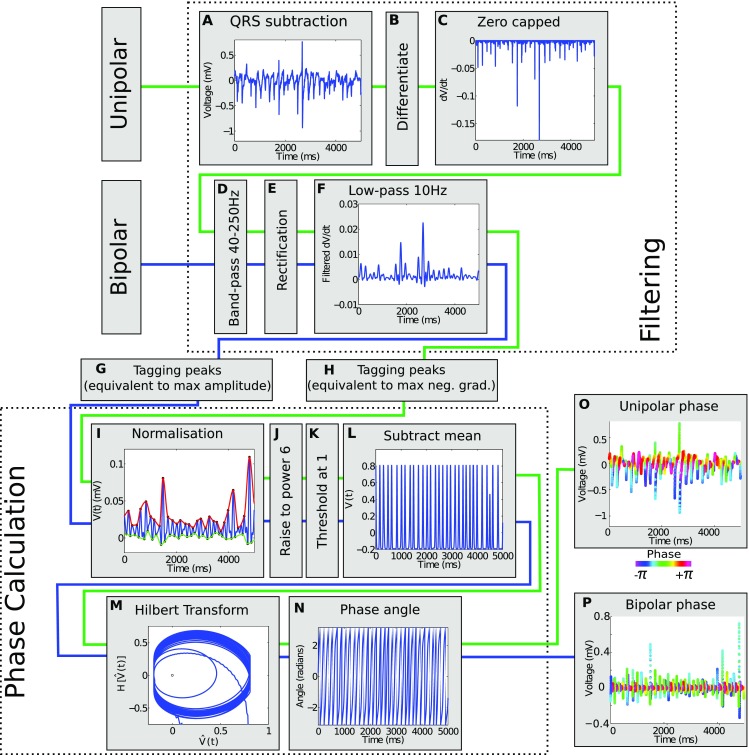
Outline of filtering and processing steps involved in phase calculation of unipolar and bipolar electrogram signals. Raw unipolar electrograms are preprocessed (a–c). Both unipolar electogram derivatives and bipolar electrograms are then pre-processed (d–f) before tagging of individual activations (g–h). Phase is then calculated identically for both modalities of signal (i–n) to give unipolar and bipolar phase (o–p).

The algorithm used for calculating phase of unipolar and bipolar signals is illustrated diagrammatically in Fig. 1, showing those steps that are common to both signal modalities and those in which unipolar and bipolar signals are treated differently. Typically, unipolar electrogram activation is defined as the location of maximum negative gradient, while bipolar activation definitions vary and include the location of maximum amplitude.^6^ With this motivation, equivalent analyses were performed on the unipolar electrogram derivative and on bipolar electrogram signals. The unipolar electrogram derivative and bipolar electrograms were filtered to enhance the sinusoidal nature of the signal and phase was then calculated. Further details on the filtering and phase calculation are outlined in the following.

#### Filtering

Unipolar electrograms obtained clinically were first pre-processed through QRS subtraction (Fig. 1a) to remove far-field ventricular depolarisation. The template subtraction technique developed by Shkurovich *et al.* was used.^32^ Other QRS subtraction techniques, including linear and cubic spline replacement methods,^1^ did not result in an appropriate differential and consequentially were not used.

For all recording modalities, a sequence of filters commonly used for dominant frequency analysis^25^ was applied to both bipolar electrograms and unipolar electrogram derivatives to emphasise the sinusoidal component of the signal and make it more suitable for phase analysis (Figs. 1d–1f). This consisted of a 40–250 Hz band-pass filter (butterworth, order 3), full-wave rectification, and a 10 Hz low-pass filter (butterworth, order 8). Filtering was performed using Matlab, using the butter and filtfilt functions to perform bidirectional processing and cancel phase shifts.^21^ For unipolar electrograms, the derivative was capped at zero before filtering was applied to prevent the selection of points of positive slope, since unipolar activation occurs at locations of steepest negative slope (Figs. 1a–1c).

#### Phase Calculation

Optimal phase calculation using the Hilbert transform requires a sinusoidal signal with zero-mean. To correctly assign each electrogram activation as a separate phase loop trajectory, activation times should be located at a constant value in the normalised signal. To achieve this, an adaptation of the pseudo empirical mode decomposition technique of Bray and Wikswo^3^ was used as follows.

Maxima in the filtered signal were tagged using a moving window of length equal to 90% of the average cycle length (estimated using the median dominant frequency), and minima sought between each pair of maxima (Figs. 1g–1h). Cubic splines were computed through each of the set of maxima and the set of minima to determine a moving upper and lower bound which was subsequently used to normalise the signal (Fig. 1i). The signal was then raised to the power of six to dampen low-amplitude untagged deflections and ensure they do not significantly contribute to the computed phase angle (Fig. 1j). The sensitivity of the algorithm to the window length for maxima selection and exponent for normalisation are presented in the supplementary material. The normalised signal was capped at one to correct portions of the signal that had exceeded the maxima line. Finally a straight mean was removed from this signal, and the Hilbert transform calculated to produce a phase-space plot and corresponding phase angle (Figs. 1l–1n). Examples of the original signals, coloured by phase angle, are shown in Figs. 1o–1p.

#### Phase Interpolation

To interpolate phase in two dimensions, locations of the twenty unipolar electrodes in each catheter were mapped to the two-dimensional representation that optimally preserved the geodesic distances between points^29^; bipolar electrode locations in two dimensions were assigned to be at the mid-points of the constituent unipolar electrode locations. Two-dimensional phase maps were generated by spatial interpolation of phase angle recordings to a regular grid with 2 mm spacing, using an exponential mapping^19^ and cubic interpolation (see supplementary material for the effects of interpolation method). Phase singularities were identified as locations where the phase was undefined, and, upon winding around the point, the phase changes by $2\pi$, meaning it is a site of non-zero topological charge.^4^ For each catheter recording location, the number of phase singularities was calculated for each frame in the recording, and then averaged over all frames to calculate the mean and standard deviation number of phase singularities expected at any time instance.

### Correlation Between Data Modalities

#### Phase Correlation

To compare the spatial distribution of phase angle values calculated using bipolar electrogram phase to unipolar electrogram phase, the correlation between the corresponding spatial phase maps were calculated for each temporal sample. A two-dimensional circular correlation measure suitable for angular data was used, following Jammalamadaka and Sengupta^19^ and available on the Matlab File exchange.^2^ The formula is given by

$$\begin{aligned} r = \frac{\sum _{m}\sum _{n}\sin {(A_{mn}-\bar{A})}\,\sin {(B_{mn} - \bar{B})}}{\sqrt{\sum _{m}\sum _{n}\sin ^2{(A_{mn}-\bar{A})}\, \sin ^2{(B_{mn}-\bar{B})} }}, \end{aligned}$$

where *A* and *B* are the two phase distributions and $\bar{A}$ and $\bar{B}$ are their respective means, calculated using an exponential mapping. As with the linear correlation coefficient, the circular correlation coefficient takes values between −1 and 1, and a perfect correlation of 1 is obtained if the two-dimensional distributions are identical modulo $2\pi$.

The difference between unipolar and bipolar phase was also calculated as the sum over the grid of the absolute difference between phase images over time (see supplementary material).

#### Wavefront Direction

For AT electrogram data, wavefront directions determined using local activation times from the clinical system or from phase were first calculated in two dimensions (see "Phase Interpolation" section) and then mapped to three dimensions. For each of the thirty catheters analysed, conduction velocity was estimated using the local activation times assigned by the electroanatomic mapping system and the mapped two-dimensional electrode locations. To calculate conduction velocity, the equation for a propagating circular wave measured at these locations was fitted to the activation times.^27^

**Figure 2:**
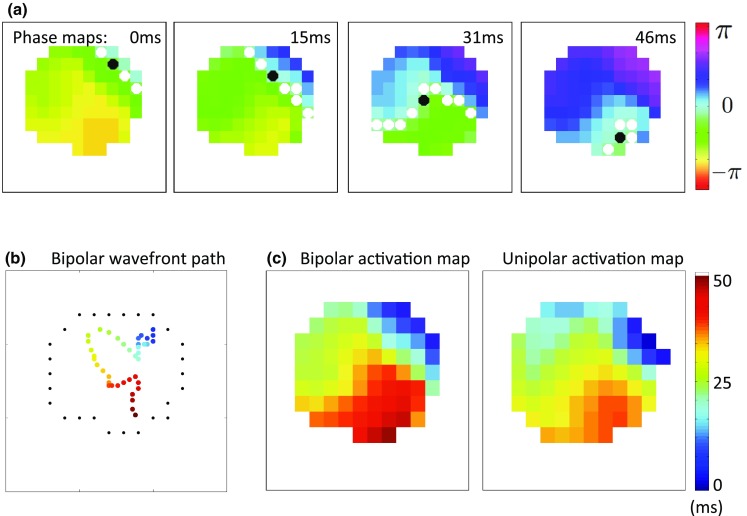
Methodology used to determine activation maps and wavefront direction from phase. (a) Isophase maps with lines of isophase 0 (white dots). Mid-point of each wavefront is indicated as a black dot. (b) Mid-points are joined and smoothed to mark wavefront path over time from blue to red. (c) Times corresponding to the start and end of this path define the time window for constructing activation time maps for bipolar and unipolar phase.

For AT phase maps, the conduction velocity vector was computed as follows. Wavefronts were defined as lines of zero phase of at least three connected pixels, as shown in Fig. 2. Waves were tracked over time subject to a similarity threshold, allowing multiple wavefronts to coexist in any given frame. Propagating wavefronts in the current frame were matched with those in the following frame if the number of matching pixels in the wavefront was greater than 10% of the number of pixels in the wavefront. Mid-points of each wavefront were joined as a trajectory if they belong to the same wave; the path of these mid-points indicates the approximate direction of the wavefront over time. For AT wavefront direction analyses, only those wavefronts for which the trajectory achieved a satisfactory fit to a straight line were retained according to a least squares residual threshold. The conduction velocity vector was then defined by the vector from one- to three-quarters along the original trajectory (to avoid edge effects) and mapped back to the three-dimensional geometry.^29^

#### Activation Time Maps

To calculate activation time maps during AF, the methodology outlined in the “Wavefront Direction” section was used to select suitable time windows. For AF data, wavefront direction was not required to be planar. Windows of AF activity that contain wavefronts that last at least 25 ms were identified, and the start and end times of each wavefront gave a time window to construct activation time maps from unipolar and bipolar phase. These time windows were selected based on wavefronts detected in the bipolar electrogram phase since bipolar electrogram phase was generally less noisy than unipolar electrogram phase. The threshold of 25 ms was chosen with the motivation that an AF wavefront travelling at approximately 0.4 m s^−1^ would cover half of an Afocus catheter in this time. This was repeated for all wavefronts and the correlations between each pair of activation time maps were calculated.

## Results

### Simulated Electrograms

**Figure 3:**
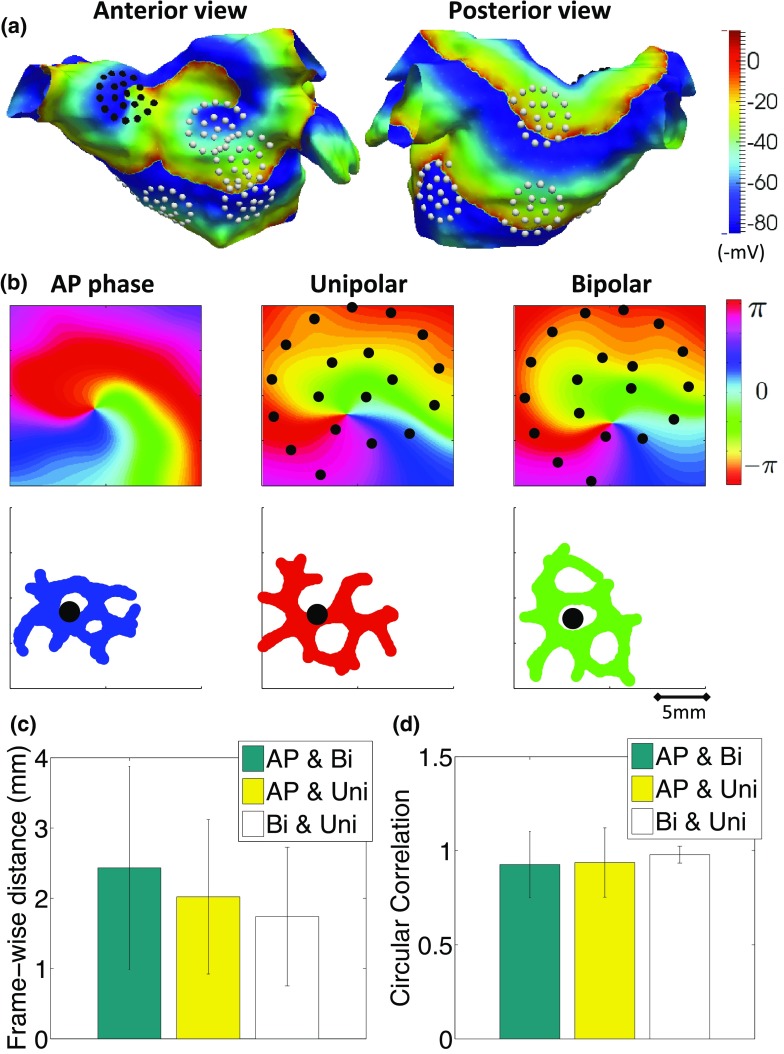
Comparison of unipolar and bipolar phase to action potential phase in simulated data. (a) Transmembrane potential map with distribution of catheters positions analysed (black and white dots). A rotor meanders in the vicinity of the catheter location shown in black on the anterior wall. These electrode locations were projected to two dimensions using a geodesic flattening, and phase maps calculated based on high-resolution action potential phase, and unipolar and bipolar electrogram phase at the AFocus electrode locations. (b) Phase maps (top) and rotor trajectories (bottom, centre indicated as black dot) for black catheter on anterior wall in (a). Projected unipolar electrode locations are indicated by the black dots in the top row, and bipolar recordings at the mid-points of paired unipoles. (c) Mean frame-wise differences between rotor-core measured using different data types were small (<4 mm diameter of an ablation catheter). (d) Mean circular correlation coefficients measured across nine catheters are high.

The phase mapping algorithm was first validated using simulated electrogram data. Fig. 3a shows an anterior and posterior view of a snapshot of transmembrane potential from simulated fibrillation, with electrode locations of the nine catheters marked. Fig. 3b shows example phase maps, along with the rotor-core trajectories for simulated AP, unipolar electrogram and bipolar electrogram phase. The rotor trajectories are observed to have a similar spread, but are affected by the locations of the recordings (phase singularities meander between recording locations). Unipolar and bipolar trajectories are more similar to each other than to the AP trajectory because the nineteen bipolar recording points are mid-way between pairs of the twenty unipolar recording points. The time-averaged location of phase singularities were close together (distance between AP and bipolar centre 0.76 mm; AP and unipolar centre 0.53 mm; bipolar and unipolar centre 0.59 mm). Frame-wise differences were also small (Fig. 3c), and less than the 4mm diameter of an ablation catheter. Median circular correlation coefficients were high across the nine catheters (Fig. 3d), showing that the calculated phase agree well between action potential, unipolar electrogram and bipolar electrogram data.

### Micro-electrode Array (MEA) Electrograms

**Figure 4:**
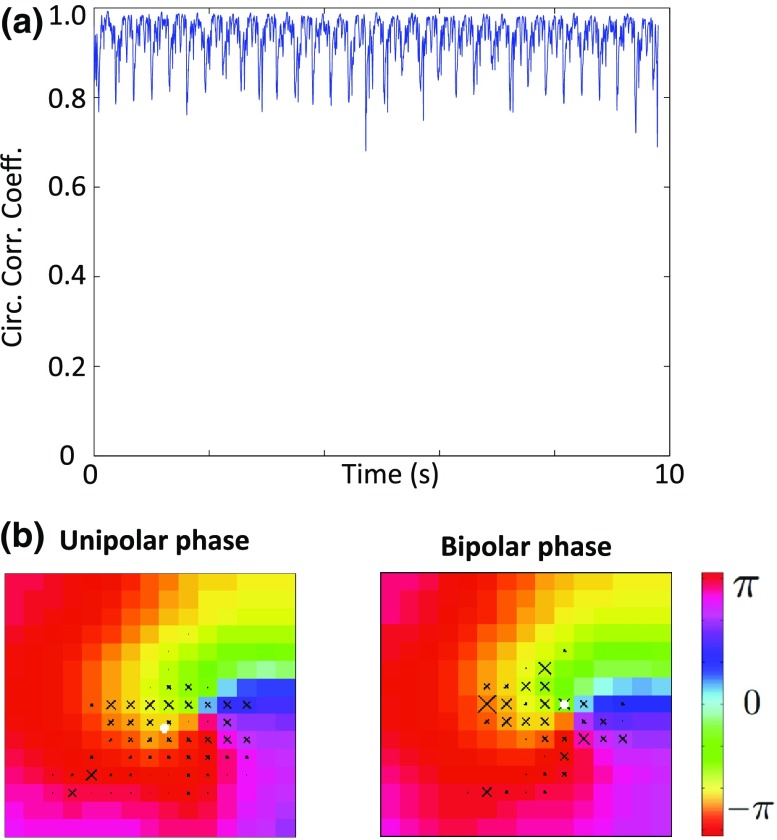
MEA unipolar phase and bipolar phase lead to similar phase singularity locations. (a) The circular correlation coefficient between unipolar and bipolar phase maps measured across an electrode array varies over time but is generally high, with a minimum value of 0.66 and a median of 0.95. (b) Phase singularity locations are indicated by a cross (magnitude indicates the number of occurrences at that location); white dot shows centre of trajectory. The domain size is 1.4 × 1.4 mm^2^.

A ten-second MEA recording was analysed for which there was a single meandering rotor within the recording area. Phase singularity locations calculated using unipolar and bipolar phase mapping are shown in Fig. 4, in which it can be seen that the rotor core trajectory covers a similar area for each modality. Quantitatively, the mean difference on a frame-by-frame basis of the rotor core location measured using unipolar or bipolar electrogram phase is 210 ± 173 *μ*m, and the difference in the location of the centre of mass of the trajectory is 153 *μ*m (inter-electrode distance 200 *μ*m). The median circular correlation coefficient between the phase maps for the electrode array measured using unipolar and bipolar electrogram phase is 0.95, showing unipolar phase is highly correlated with bipolar phase.

### Validation Using Atrial Tachycardia Data

**Figure 5:**
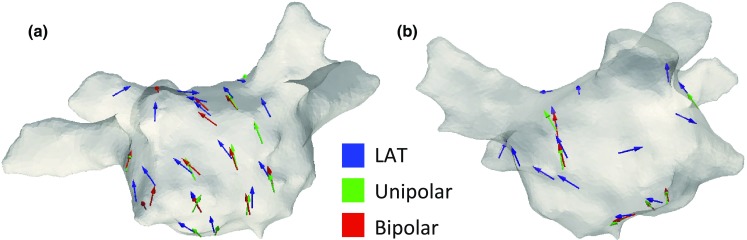
Clinical AT phase mapping direction validation. Directions measured using voltage (activation times, blue, gold standard), unipolar electrogram phase (green) and bipolar electrogram phase (red) show a similar global pattern; posterior view (a), anterior view (b).

The wavefront directions calculated using activation times exported from the electro-anatomic mapping system (30 catheter locations), using bipolar phase (18 catheter locations) and using unipolar phase (15 catheter locations) are plotted in Fig. 5, in which it can be seen that the global activation patterns calculated using the different measures agree well on the posterior wall since arrows are seen to overlap and follow the same pattern. Directions were calculated from the unipolar phase of multiple beats of AT (99.9 ± 27.7 beats per catheter recording location) or the bipolar phase of multiple beats of AT (88.9 ± 19.7 beats per catheter recording location).

### Number of Phase Singularities

**Figure 6:**
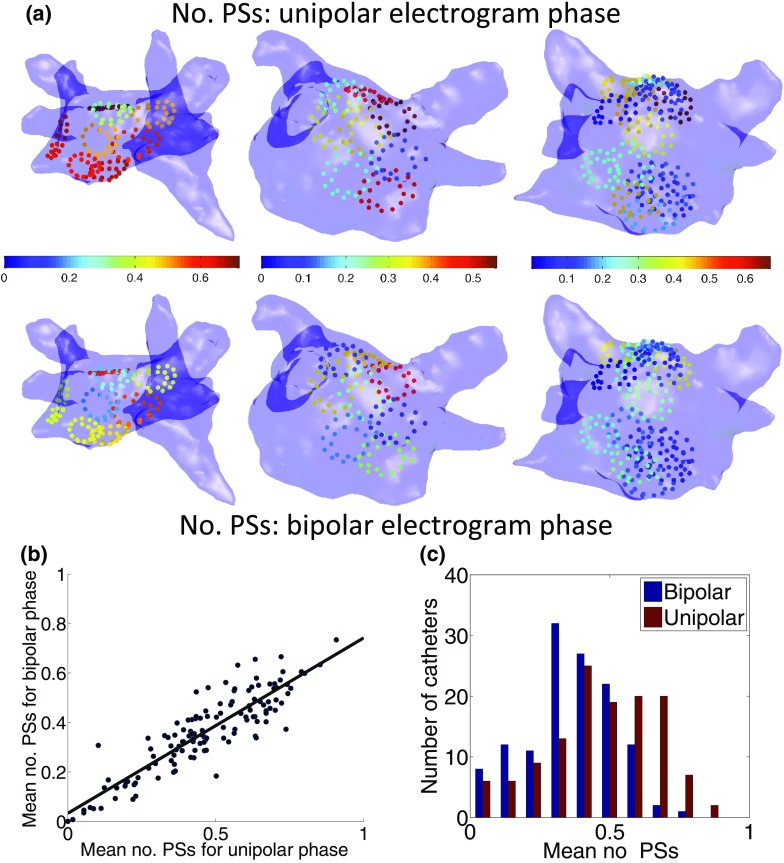
Regions of high and low phase singularity content derived from unipolar and bipolar phase maps for clinical data agree well. (a) Average number of phase singularities (PSs) calculated over time is shown per catheter recording position for both unipolar (top row) and bipolar (bottom row) phase for three patients. (b) Scatter plot showing mean number of PSs measured using bipolar phase against the mean number for unipolar phase (*n* = 127; line of best fit is: $y = 0.03+0.71x$, with $R^2 =$ 0.79). (c) Histogram to show the distribution of mean number of phase singularities across the different catheter placements (*n* = 127), for bipolar and unipolar phase. The mean number of PSs represents the expected number at any time instance. Recording durations were 16–106 s with a mean of 34 s, sampled at 2.0345 kHz.

Figure 6 shows the number of phase singularities per catheter recording position for three patients: there is visually a good correspondence between the ranking of catheter recordings using this measure for unipolar and bipolar electrograms. This was quantified across eleven patients (6–17 catheter locations per patient; range of durations 16–106 s) in terms of the relationship between the average number of phase singularities per frame for each catheter recording for unipolar phase and bipolar phase (Figs. 6b, 6c), for which the correlation coefficient was 0.89. Overall, categorising catheter recording positions by the number of phase singularities, indicating rotational content, was independent of data type. The mean number of phase singularities across all 127 catheter recordings was 0.36 ± 0.16 per catheter recording position for bipolar electrogram phase and 0.47 ± 0.20 per catheter recording for unipolar electrogram phase. The mean of the differences in the number measured using each datatype per catheter recording position was −0.10 ± 0.09 (bipolar minus unipolar).

### Phase Angle and Activation Time Map Correlation

**Figure 7:**
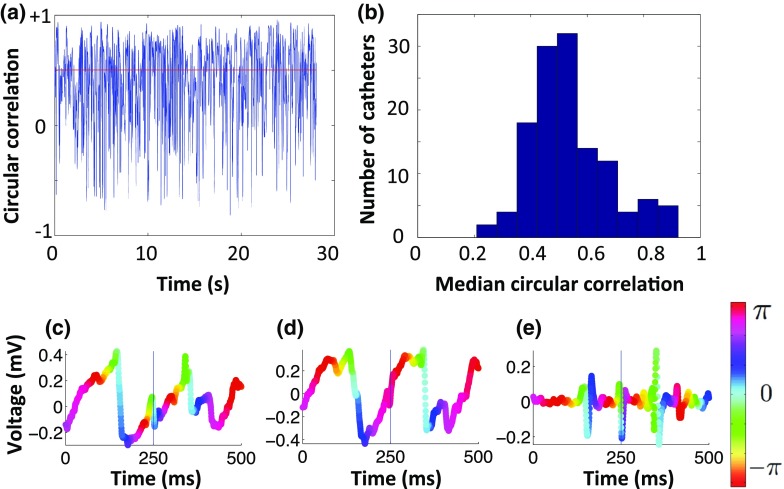
During human AF, low phase angle correlation may occur due to differences in activation assignment. (a) Plot of circular correlation coefficient between the unipolar and bipolar phase angles for an example catheter, with the median value (0.50) shown by the red line. (b) Histogram showing the distribution of median circular correlation coefficient measured between bipolar and unipolar phase for each of the 127 catheter recordings. (c) Unipolar electrogram coloured by phase angle; (d) paired unipolar electrogram with a missed activation at time 250 ms; (e) corresponding bipolar electrogram coloured by phase in which an activation is seen at 250 ms. The difference in activation assignment at 250 ms leads to a low correlation between unipolar and bipolar phase maps.

Figure 7a shows an example plot of phase angle circular correlation between unipolar and bipolar phase for one catheter recording. This measures whether the phase values calculated using unipolar and bipolar electrograms are correlated over the duration of the recording. The correlation shows the activity varies in consistency between the data types over time. For this analysis, the median correlation per catheter is considered so as not to give undue weight to the low correlation time segments.

The median correlation per catheter are shown as a histogram plot across the 127 catheters in Fig. 7b; there is a wide range of correlations (0.21–0.92). This indicates that catheters show different degrees of agreement, depending on the amount of fractionation within the signal, whether there are wavefront collisions and how planar the wave is. The median of the median correlations is 0.51, indicating that half the catheters had a median correlation above a correlation threshold of 0.5. The maximum *p* value across the catheters was 0.02 (mean 5.29 × 10^−4^), showing a statistically significant relationship between unipolar and bipolar phase.

As seen in Fig. 7a, there were time segments for which unipolar and bipolar phase correlated poorly (10.4% of the recording has a circular correlation coefficient below 0, while 49.7% has one below a threshold of 0.5 circular correlation). These were investigated in more detail to identify common occurrences leading to differences. Some instances of low correlation were due to a missed activation on the unipolar or bipolar electrogram. An example is shown in Figs. 7c–7e, for which a likely activation in the second unipolar electrogram was missed. This example is difficult to assign in the absence of information from other unipolar and bipolar electrograms. Other instances were due to a prolonged unipolar downstroke; in which case it was difficult to determine activation time.

**Figure 8:**
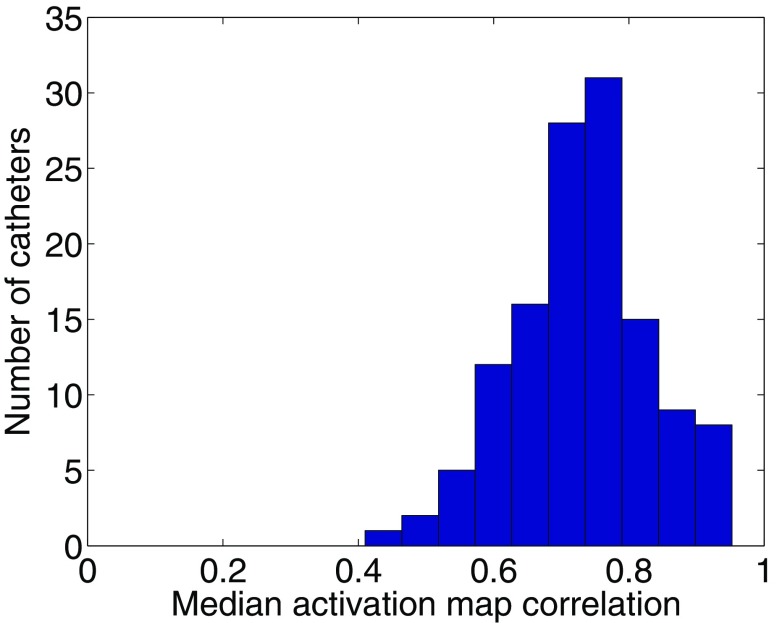
Histogram to show the median correlation coefficient measured between clinical AF activation maps from bipolar and unipolar phase, demonstrating that the correlation is approximately normally distributed. The overall median is 0.73.

Activation time maps indicate wavefront propagation so a further measure of the correspondence between unipolar and bipolar phase results is whether there is agreement between maps of activation time constructed from timings of constant phase. A histogram to show the median correlation for each catheter is shown in Fig. 8, where the overall median is 0.73.

## Discussion

Methods were presented for phase mapping of unipolar electrogram and bipolar electrogram signals, which were tested on simulated data, and subsequently applied to experimental and clinical electrograms. Unipolar and bipolar phase were seen to perform similarly to the reference action potential phase in simulated data, and agreed well with each other in experimental data, suggesting the method faithfully reproduces the activation patterns. Clinical atrial tachycardia data provided validation of wavefront directions measured from bipolar and unipolar phase against those recorded by an electroanatomic mapping system. In clinical atrial fibrillation data, phase calculated from unipolar and bipolar signals did not exhibit as strong a correlation as for the other datatypes, which is expected as the clinical signals are more complex.

### Atrial Tachycardia Validation Studies

Clinical electrogram data exhibit a higher degree of complexity than simulated electrograms and *in vitro* electrograms, and as such a test to check whether the developed algorithms are appropriate for clinical data was performed using AT data in the “Validation Using Atrial Tachycardia Data” section. These data have the advantage that activation times and patterns can be assigned confidently using voltage, for comparison with the equivalent measures from phase. Directions calculated from tracking unipolar or bipolar wavefronts using the calculated phase displayed a similar global activation pattern to directions calculated using the local activation times exported from the Velocity electro-anatomic system (Fig. 5), which provides a gold standard to compare the phase algorithms to. This validates the use of phase for assigning wavefront direction.

AF data offers the additional challenge of multi-component electrograms for which it is not always clear which deflections represent true activations, and so AT validation does not offer validation in the case of AF data.

### Atrial Fibrillation Analysis

The average number of phase singularities measured using unipolar and bipolar phase during AF matched well on a catheter recording by catheter recording basis ("Number of Phase Singularities" section; Fig. 6), showing that either method can be used for classifying areas by phase singularity content. This is important because clinical ablation strategies may target areas of high phase singularity density.^23^ As shown in Fig. 6, the number of phase singularities exhibited a difference between catheter recording positions, indicating there is spatial variation in rotational content across catheter recordings, which can be used to identify areas of high rotational content. Pooling data across all patients and all catheter locations allows comparison of the phase singularity density calculated for unipolar and bipolar phase, without the requirement of classifying the nature of the reentrant activity a priori. The circular correlation coefficient for the phase angle maps shows that the situation is more complicated, as this measure varies in time for each catheter recording location, and there are time segments for which unipolar and bipolar phase do not correlate well (Fig. 7). There are also differences in the median correlation coefficient measured for each catheter recording location, with some catheters recordings showing a higher correspondence than others. These catheter positions were observed to be those for which more of the wavefronts were planar, and consequently the phase was easier to assign. Further dissection of these differences in terms of the underlying activity warrants further investigation.

Activation time maps correlated well during AF ("Phase Angle and Activation Time Map Correlation" section; Fig. 8), showing that the wavefront propagation patterns detected using unipolar and bipolar phase were well correlated, so that both unipolar and bipolar phase measure corresponding activation wavefronts, even during fibrillation. One limitation of the method used to correlate activation time maps was that activation windows for the construction of the maps were based on wavefronts detected in bipolar phase that last at least 25 ms. This technique circumvents the challenge of automatically choosing suitable activation windows during AF; but will exclude wavefronts that have a short duration perhaps due to collision, and might also exclude time segments in which the phase is more noisy and wavefront patterns are unclear. Excluding these wavefronts could bias the correlation score.

Time segments for which unipolar and bipolar phase did not correlate well were investigated on an individual basis. Although the differences were occasionally due to a missed unipolar or bipolar deflection (see Figs.  7c–7e), for the majority of cases in which there were differences it was difficult to determine which of the unipolar or bipolar phases predicted the true activation times more closely.

### Comparison of Simulated, Experimental and Clinical Electrogram and Action Potential Phase Calculations

Whereas action potential phase captures both depolarisation and repolarisation as separate stages of the action potential, electrogram phase indicates activations during AF and then progression within the cycle between activations. This is because stages of repolarisation are more difficult to identify for electrograms during AF. Thus phase mapping can be viewed as a means to indicate propagation of the activation wavefront only. Filters are required to make the electrogram data more sinusoidal so that phase analysis can be applied.

The sequence of filters used in this study are commonly used for analysing bipolar electrograms. There are studies that investigate how these filters affect the bipolar electrogram and its frequency content.^8^ However, the application of these filters prior to phase analysis is novel, to the best of our knowledge. The filtering process used on the unipolar signal does not alter the estimated activation times since firstly it is done on the gradient rather than on the amplitude and peaks are preserved in the filtering process, meaning the predicted activation times are not altered. Further, capping above at zero (Fig. 1c) had no effect as it is only the negative peaks that are used as estimated activations. This reasoning allowed the development of a new unipolar phase calculation method that applied the same steps as used to find phase from the amplitude of the bipolar signal, except that the derivative of the QRS-subtracted unipolar signal was used instead.

### Comparison with Other Methodologies for Calculating Phase

There are multiple clinical studies that use phase to assess rotational activity, including Haissaguerre *et al.* who use unipolar phase for identifying rotational activity from electrocardiographic imaging data,^18^ and Narayan *et al.* who use phase to identify rotors using a basket catheter.^23^ However, to the best of our knowledge, there are currently no published methodologies for calculating bipolar electrogram phase.

Nash *et al.* 24 analysed human VF dynamics using phase calculated from unipolar electrograms measured using an epicardial sock consisting of 256 electrodes. They use a quadratic detrending technique to ensure that signals have mean zero, with activation times at voltage zero. This technique works well for unipolar electrograms during VF; however, this technique is not expected to perform as well on unipolar AF electrograms due to the lower signal to noise ratio, meaning that it may be difficult to distinguish activations from baseline noise.^20^ In addition, Umapathy *et al.* 33 provide an extensive review of phase mapping techniques for electrogram data, although no methodology specific to atrial electrograms are presented.

In our study, problems with incorrect phase assignment between beats were not observed, in contrast to previous studies.^26^ This is because the filtering and normalisation steps of our algorithm led to a sinusoidal signal of suitable amplitude and mean.

Similar to the approach of Kuklik *et al.* 20 where wavelets were only generated where there is a negative gradient, the gradient of unipolar electrograms was capped at zero, before filtering, as this also prevented the algorithm from assigning activation at locations of steep positive gradient.

For the method of Kuklik *et al.*,^20^ the period of each sinusoidal wavelet is equal to the mean of the cycle length of the signal. Their method has the disadvantage that it may not work well in the situation of varying cycle length. The method developed in this paper also uses a window length to tag deflections and so may suffer similar limitations. A combination of window length and amplitude may be more appropriate, and an advantage of the method developed in this paper over the method of Kuklik *et al.* is that our methodology can be easily modified to use other methods of activation tagging.

In contrast to the method of Kuklik *et al.*,^20^ the methodology presented here creates a more sinusoidal signal for phase analysis by applying a sequence of filters commonly used in dominant frequency analysis. A zero-mean signal with suitable activation times is then defined using an approach similar to the method of Bray and Wikswo for calculating action potential phase,^3^ with an additional series of steps to ensure noise does not affect phase angle (see Supplementary material S-Figs. 1, 2).

The method developed here shows a reasonably close correspondence between unipolar and bipolar phase, because the same sequence of filters and phase calculation techniques were applied to the bipolar electrogram that were applied to the derivative of the unipolar signal. Thus the methodology works for both unipolar and bipolar electrograms from simulation, experimental or clinical recordings.

### Limitations and Future Work

One potential technique to improve the method would be to use the unipolar and bipolar phase in conjunction, such that time segments in which the unipolar and bipolar phase agree are interpreted to represent correctly tagged deflections, and times for which they disagree indicate that at least one of the electrograms is incorrectly tagged. Bipolar electrograms were observed to be less noisy than unipolar electrograms, and thus bipolar phase is hypothesised to give a more reliable representation of the wavefront dynamics. Unipolar electrograms could then be used to define the activation time of an identified complex more accurately. The application of the method to fractionated electrograms should be compared to times chosen by an experienced clinician, which we leave for future work.

### Conclusions

To our knowledge, we have presented the first published methodology appropriate for calculating phase of both unipolar and bipolar electrogram data from simulation, experimental or clinical recordings. The algorithm is validated against action potential phase using simulated data; between experimental multi-electrode array unipolar and bipolar phase; and for wavefront identification in clinical atrial tachycardia recordings. It is appropriate for determining activation patterns and assessing rotational content of AF electrogram data. The algorithm is robust for clinical recordings, laboratory measurements and simulated electrograms, and enables accurate quantification of AF wavefronts and sources from sequential mapping data. This is significant because identifying critical sources, such as rotors, in AF, allows more accurate targeting of ablation therapy and improved patient outcomes.

## Electronic supplementary material

Below is the link to the electronic supplementary material.

Supplementary material 1 (PDF 6660 KB)

## Abbreviations

AF: Atrial fibrillation
AP: Action potential
AT: Atrial tachycardia
MEA: Micro electrode array
PS: Phase singularity
VF: Ventricular fibrillation

## References

[1] Ahmad, a., J. L. Salinet, P. Brown, J. H. Tuan, P. Stafford, G. A. Ng, and F. S. Schlindwein. QRS subtraction for atrial electrograms: flat, linear and spline interpolations. *Med. Biol. Eng. Comput.* 49:1321–1328, 2011. 10.1007/s11517-011-0829-9 21959592

[2] Berens, P. Circular Statistics Toolbox. http://www.mathworks.com/matlabcentral/fileexchange/10676-circular-statistics-toolbox--directional-statistics-/content/circ_corrcc.m

[3] Bray M-A, Wikswo J (2002). Considerations in phase plane analysis for nonstationary reentrant cardiac behavior. Physical Review E.

[4] Bray M-A, Wikswo JP (2002). Use of topological charge to determine filament location and dynamics in a numerical model of scroll wave activity. IEEE transactions on bio-medical engineering.

[5] Cantwell, C., D. Moxey, A. Comerford, A. Bolis, G. Rocco, G. Mengaldo, D. D. Grazia, S. Yakovlev, J.-E. Lombard, D. Ekelschot, B. Jordi, H. Xu, Y. Mohamied, C. Eskilsson, B. Nelson, P. Vos, C. Biotto, R. Kirby, and S. Sherwin. Nektar++: an open-source spectral/hp element framework. *Comput. Phys. Commun.* 192:205–219, 2015.

[6] Cantwell C, Roney C, Ng F, Siggers J, Sherwin S, Peters N (2015). Techniques for automated local activation time annotation and conduction velocity estimation in cardiac mapping. Computers in biology and medicine.

[7] Cantwell CD, Yakovlev S, Kirby RM, Peters NS, Sherwin SJ (2014). High-order spectral/hp element discretisation for reaction-diffusion problems on surfaces: Application to cardiac electrophysiology. Journal of Computational Physics.

[8] Castells F, Cervigón R, Millet J (2014). On the preprocessing of atrial electrograms in atrial fibrillation: understanding Botteron’s approach. Pacing and clinical electrophysiology : PACE.

[9] Clayton RH, Nash MP (2015). Analysis of cardiac fibrillation using phase mapping. Cardiac electrophysiology clinics.

[10] simplifying complexity in computational models of ventricular fibrillation (2006). Clayton, R. H., E. a. Zhuchkova, and a. V. Panfilov. Phase singularities and filaments. Progress in biophysics and molecular biology.

[11] Courtemanche M, Ramirez RJ, Nattel S (1998). Ionic mechanisms underlying human atrial action potential properties: insights from a mathematical model. American Journal of Physiology-Heart and Circulatory Physiology.

[12] Courtemanche M, Ramirez RJ, Nattel S (1999). Ionic targets for drug therapy and atrial fibrillation-induced electrical remodeling: insights from a mathematical model. Cardiovascular research.

[13] Dias P, Desplantez T, El-Harasis MA, Chowdhury RA, Ullrich ND, de Diego AC, Peters NS, Severs NJ, MacLeod KT, Dupont E (2014). Characterisation of connexin expression and electrophysiological properties in stable clones of the hl-1 myocyte cell line. PloS one.

[14] Ganesan AN, Kuklik P, Lau DH, Brooks AG, Baumert M, Lim WW, Thanigaimani S, Nayyar S, Mahajan R, Kalman JM, Roberts-Thomson KC, Sanders P (2013). Bipolar electrogram shannon entropy at sites of rotational activation: implications for ablation of atrial fibrillation. Circulation. Arrhythmia and electrophysiology.

[15] Geuzaine C, Remacle J-F (2009). Gmsh: a three-dimensional finite element mesh generator with built-in pre- and post-processing facilities. International Journal for Numerical Methods in Engineering.

[16] Gray RA, Pertsov AM, Jalife J (1998). Spatial and temporal organization during cardiac fibrillation. Nature.

[17] Haissaguerre M, Hocini M, Denis A, Shah AJ, Komatsu Y, Yamashita S, Daly M, Amraoui S, Zellerhoff S, Picat M-Q (2014). Driver domains in persistent atrial fibrillation. Circulation.

[18] Haissaguerre M, Hocini M, Shah AJ, Derval N, Sacher F, Jais P, Dubois R (2013). Noninvasive panoramic mapping of human atrial fibrillation mechanisms: a feasibility report. Journal of cardiovascular electrophysiology.

[19] Jammalamadaka, S. R. and A. Sengupta. Topics in Circular Statistics, vol. 6. River Edge: World Scientific 2001, p. e20505.

[20] Kuklik P, Zeemering S, Maesen B, Maessen J, Crijns HJ, Verheule S, Ganesan AN, Schotten U (2015). Reconstruction of instantaneous phase of unipolar atrial contact electrogram using a concept of sinusoidal recomposition and hilbert transform. IEEE transactions on biomedical engineering.

[21] Laughner JI, Ng FS, Sulkin MS, Arthur RM, Efimov IR (2012). Processing and analysis of cardiac optical mapping data obtained with potentiometric dyes. American Journal of Physiology-Heart and Circulatory Physiology.

[22] Nademanee K, McKenzie J, Kosar E, Schwab M, Sunsaneewitayakul B, Vasavakul T, Khunnawat C, Ngarmukos T (2004). A new approach for catheter ablation of atrial fibrillation: mapping of the electrophysiologic substrate. Journal of the American College of Cardiology.

[23] Narayan SM, Baykaner T, Clopton P, Schricker A, Lalani GG, Krummen DE, Shivkumar K, Miller JM (2014). Ablation of rotor and focal sources reduces late recurrence of atrial fibrillation compared with trigger ablation alone: extended follow-up of the CONFIRM trial (Conventional Ablation for Atrial Fibrillation With or Without Focal Impulse and Rotor Modulat. Journal of the American College of Cardiology.

[24] Nash MP, Mourad A, Clayton RH, Sutton PM, Bradley CP, Hayward M, Paterson DJ, Taggart P (2006). Evidence for multiple mechanisms in human ventricular fibrillation. Circulation.

[25] Ng J, Kadish AH, Goldberger JJ (2006). Effect of electrogram characteristics on the relationship of dominant frequency to atrial activation rate in atrial fibrillation. Heart Rhythm.

[26] Pashaei A, Bayer J, Meillet V, Dubois R, Vigmond EJ (2015). Computation and Projection of Spiral Wave Trajectories During Atrial Fibrillation: A Computational Study. Cardiac Electrophysiology Clinics.

[27] Roney, C. H., C. D. Cantwell, N. A. Qureshi, R. L. Ali, E. T. Y. Chang, P. B. Lim, S. J. Sherwin, N. S. Peters, J. H. Siggers, and F. S. Ng. An automated algorithm for determining conduction velocity, wavefront direction and origin of focal cardiac arrhythmias using a multipolar catheter. In: 36th Annual International Conference of the IEEE Engineering in Medicine and Biology Society, 2014, pp. 1583–1586. 10.1109/EMBC.2014.6943906 25570274

[28] Roney, C. H., C. D. Cantwell, J. H. Siggers, F. S. Ng, and N. S. Peters. A novel method for rotor tracking using bipolar electrogram phase. In: Computing in Cardiology (CinC), 2014, pp. 233–236.

[29] Roney, C. H., K. N. Tzortzis, C. D. Cantwell, N. A. Qureshi, R. L. Ali, P. B. Lim, J. H. Siggers, F. S. Ng, and N. S. Peters. A technique for visualising three-dimensional left atrial cardiac activation data in two dimensions with minimal distance distortion. In: 37th Annual International Conference of the IEEE Engineering in Medicine and Biology Society, 2015, pp. 7296–7299. 10.1109/EMBC.2015.7320076 26737976

[30] Sanders P, Berenfeld O, Hocini M, Jaïs P, Vaidyanathan R, Hsu L-F, Garrigue S, Takahashi Y, Rotter M, Sacher F, Scavée C, Ploutz-Snyder R, Jalife J, Haïssaguerre M (2005). Spectral analysis identifies sites of high-frequency activity maintaining atrial fibrillation in humans. Circulation.

[31] Sato D, Xie L-H, Sovari AA, Tran DX, Morita N, Xie F, Karagueuzian H, Garfinkel A, Weiss JN, Qu Z (2009). Synchronization of chaotic early afterdepolarizations in the genesis of cardiac arrhythmias. Proceedings of the National Academy of Sciences of the United States of America.

[32] Shkurovich, S., A. V. Sahakian, and S. Swiryn. Detection of atrial activity from high-voltage leads of implantable ventricular defibrillators using a cancellation technique. *IEEE Trans. Bio-Med. Eng.* 45:229–334, 1998. 10.1109/10.661270 9473845

[33] Umapathy K, Nair K, Masse S, Krishnan S, Rogers J, Nash MP, Nanthakumar K (2010). Phase mapping of cardiac fibrillation. Circulation. Arrhythmia and electrophysiology.

[34] Yushkevich PA, Piven J, Hazlett HC, Smith RG, Ho S, Gee JC, Gerig G (2006). User-guided 3D active contour segmentation of anatomical structures: Significantly improved efficiency and reliability. NeuroImage.

