# Supplementary material for: Rotor Tracking Using Phase of Electrograms Recorded During Atrial Fibrillation
Source: Ann Biomed Eng. 2016 Dec 5;45(4):910–23. doi: 10.1007/s10439-016-1766-4 (PMC5362653; doi:10.1007/s10439-016-1766-4)
Supplement: Supplementary file 1 — Supplementary material 1 (PDF 6660 KB) [file 10439_2016_1766_MOESM1_ESM.pdf]

Supplementary material for: Rotor Tracking Using Phase of Electrograms Recorded During Atrial Fibrillation

Caroline H. Roney, Chris D. Cantwell, Norman A. Qureshi, Rasheda A. Chowdhury, Emmanuel Dupont, Phang Boon Lim, Edward J. Vigmond, Jennifer H. Tweedy, Fu Siong Ng, Nicholas S. Peters

MEA phase analysis:

The following modifications were made to the clinical phase mapping algorithm for the analysis of MEA electrograms. In the case of MEA unipolar electrograms, the derivative signal contained a large amount of noise, and thus to avoid selecting multiple minima of the derivative signal, windows were created, which were the regions between the minima of the original electrogram signal (notch filtered at 50 Hz), and only one minimum of the derivative signal was chosen per window.

In addition, noisy electrograms were automatically excluded from phase interpolation by identifying the number of activations within the phase signal (where activations occur at phase $\pi/2$) and excluding a signal if the number of activations was above a threshold chosen based on the dominant frequency of the activity.

Sensitivity analysis methods:

To determine suitable parameters for the window length used in tagging maxima within the signal and the exponent used for normalization of the signal (steps shown in Fig1 of the main manuscript), a sensitivity analysis was performed. This was first performed using the in silico data described in the "Simulated Data" section of the main manuscript. To examine the effects of noise on phase calculated with these parameter choices, Gaussian noise was added to the electrograms with a standard deviation of the maximum signal amplitude (A) multipled by either 0.05 (low level of noise) or 0.2 (high level of noise). Window length and exponent choice were varied individually, and the cycle length was estimated for each choice. This analysis was performed for 180 in silico unipolar electrograms and 171 bipolar electrograms, with no noise, low noise, or high noise. To test whether the resulting phase contained a reasonable number of activations, we estimated the cycle length as the mean interval between timings of isophase 0.

The window length was expressed as a percentage of the average cycle length, which was estimated using the median of the dominant frequencies measured across all bipolar electrograms. This percentage was varied from 50% to 110%. The lower bound of 50% was chosen following Bray and Wikswo [1], who use a window length of half the estimated cycle length in order to capture double potentials in optical mapping potential data.

Signals were raised to an exponent before normalization was performed in order to dampen low-amplitude untagged deflections to prevent these from contributing to the phase angle. We tested the effects of not including this step by using an exponent of 1, up to a large degree of dampening (exponent of 8).

A similar analysis was performed for electrograms recorded from one patient, but in this case no synthetic noise was added.

The effects of the choice of interpolation technique on phase singularities detected were investigated by comparing linear and cubic interpolation.

Sensitivity analysis results:
S-Fig. 1A shows the effects of window length choice on the average cycle length for simulated unipolar and bipolar electrograms with different amounts of random noise added to the signals. In the case that no noise was added, the window length parameter had little effect on the mean cycle length. This was because there were no additional deflections in the signal of sufficient amplitude to affect the phase angle. For a low level of noise, the cycle length is observed to decrease for a window length of 60% or less. For a high level of noise, this effect was more pronounced, with a large degree of decrease in average cycle length for a window length of 70% or below.

S-Fig 1B shows the equivalent plot for clinical electrograms, in which the results are seen to be qualitatively similar to those for simulated data with a low level of noise. One explanation for this is that clinical electrograms have differing degrees of noise, but the behaviour homogenizes to a low level of noise overall. For all curves in S-Fig 1A and S-Fig 1B, at a window length of 90% the mean cycle length measured from phase is within 6% of the median cycle length estimated using dominant frequencies for simulated data, and within 1% for clinical data. As such, a window length of 90% was chosen because a window length higher than this would not be suitable for electrograms with a frequency significantly higher than the median dominant frequency measured across the population of electrograms.

S-Fig. 1C shows an example of the effects of window length choice for an in silico

electrogram with synthetic noise. For a low level of noise, a window length of 50% does not include additional deflections and so assigns phase appropriately. However, for a high level of noise many additional deflections are tagged at a window length of 50%. These deflections represent noise rather than true activations. Using a window length of 90% assigns phase correctly because maxima are tagged appropriately. The same effect is observed in clinical recordings. S-Fig 1D shows an example unipolar electrogram in which an additional activation is defined incorrectly at a window length of 50%, which is not tagged at 90%. Similarly, noise may be incorrectly assigned as deflections in bipolar electrograms (S-Fig 1D) for a window length of 50%, but not for a window length of 90%.

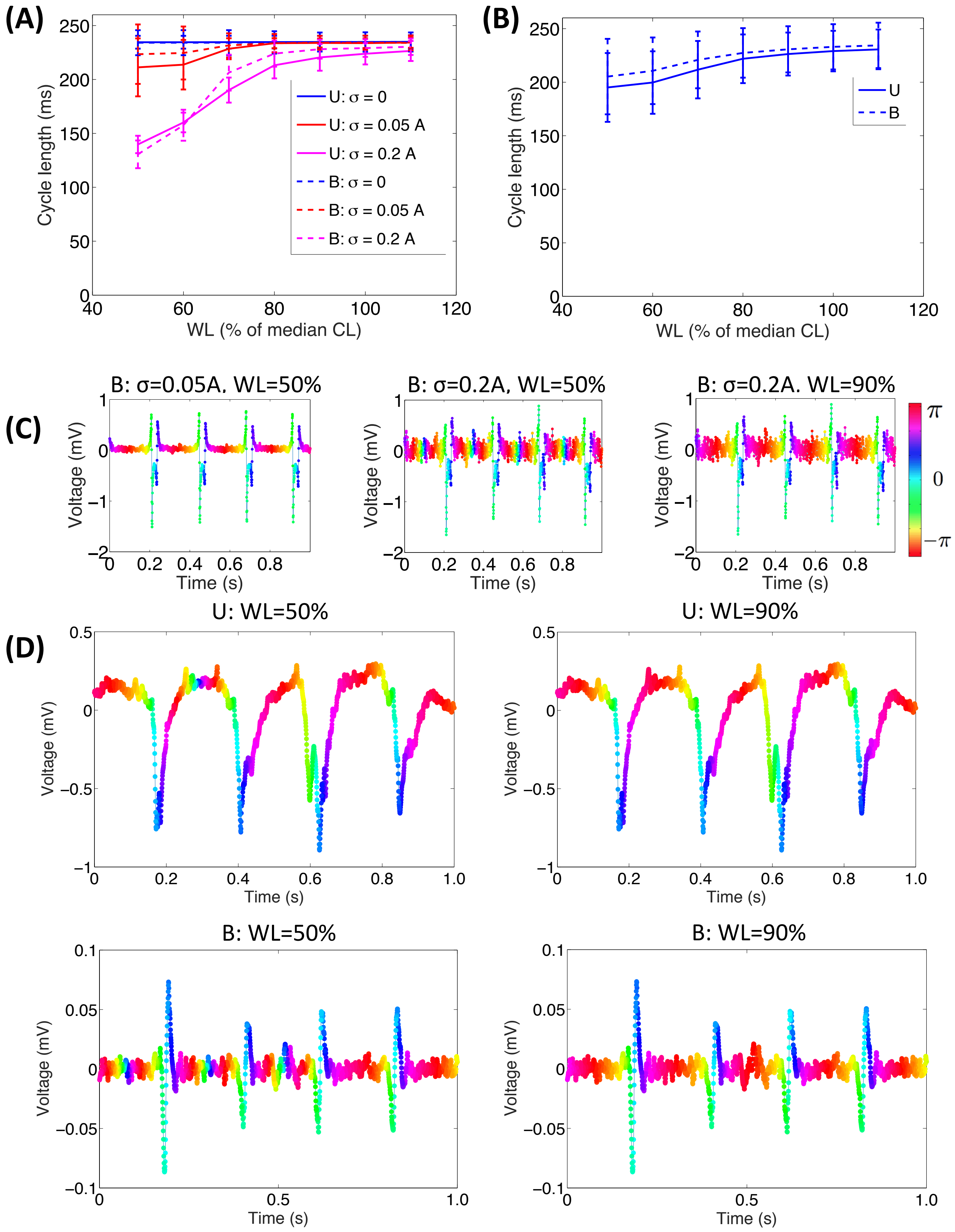

(A)
Cycle length (ms)
WL (% of median CL)
U: σ = 0
U: σ = 0.05 A
U: σ = 0.2 A
B: σ = 0
B: σ = 0.05 A
B: σ = 0.2 A
(B)
Cycle length (ms)
WL (% of median CL)
U
B
(C)
B: σ=0.05A, WL=50%
B: σ=0.2A, WL=50%
B: σ=0.2A, WL=90%
Voltage (mV)
Time (s)
π
0
−π
(D)
U: WL=50%
U: WL=90%
B: WL=50%
B: WL=90%
Voltage (mV)
Time (s)

S-Fig 1: The effects of window length for maxima tagging on phase calculation.
A) Mean and standard deviation of cycle length estimated from phase as a function of window length, for unipolar (U) and bipolar (B) in silico electrograms with different degrees of noise. Synthetic noise were added with a standard deviation (σ) equal to 0, 0.05 or 0.2 multiplied by the maximum signal amplitude (A).
B) Mean and standard deviation of cycle length estimated from phase as a function of window length, for unipolar (U) and bipolar (B) clinical electrograms for one patient in AF.
C) Electrogram phase for simulated bipolar electrograms with low noise (left) are assigned correctly at a window length of 50%, while high noise electrograms (middle) demonstrate incorrectly assigned deflections. Increasing the window length to 90% assigns the phase correctly (right).
D) For unipolar (top) and bipolar (bottom row) electrograms there are additional deflections tagged in the phase at a window length of 50% (left) that are not there at 90% (right).

The effects of exponent choice in the normalisation part of the algorithm are shown in S-Fig 2. This parameter did not have as large an effect on average cycle length compared to the effects of window length (exponent maximum range: 53ms, window length maximum range: 100ms). The exponent parameter acts to dampen low-amplitude untagged deflections, which may otherwise contribute to the phase. An example of this is shown in S-Fig 2A, in which a perturbation in phase angle is seen between activations for both unipolar and bipolar electrograms. Raising the signal to the power of six during the normalisation part of the algorithm prevents this, leading to a clean phase angle. This difference is particularly evident in the phase loops of the zero-mean signal against its Hilbert transform, shown in S-Fig 2B. Trajectories for low exponent values are of variable diameter and some do not encircle the origin. For higher exponent values, these loops are more constant in diameter, indicating a more consistent assignment of phase angle through an electrogram complex.
The same effect is seen in simulated electrograms with synthetic noise, shown in S-Fig 2C. The effect of this parameter on the phase trajectory is more pronounced as the signal to noise ratio is decreased (compare S-Fig 2C top and bottom rows). For an exponent of one, this manifests as an appropriate phase assignment for a signal with no noise, a reasonable assignment for a signal with a low level of noise (S-Fig 2D left), and an assignment that varies between complexes for a high level of noise (S-Fig 2D middle). Raising the signal to the power of six results in a consistent phase assignment between electrogram complexes for signals with even a large degree of noise (S-Fig 2D right).

As such, a window length of 90% of the estimated median cycle length was used for maxima tagging, and an exponent of six for normalisation.

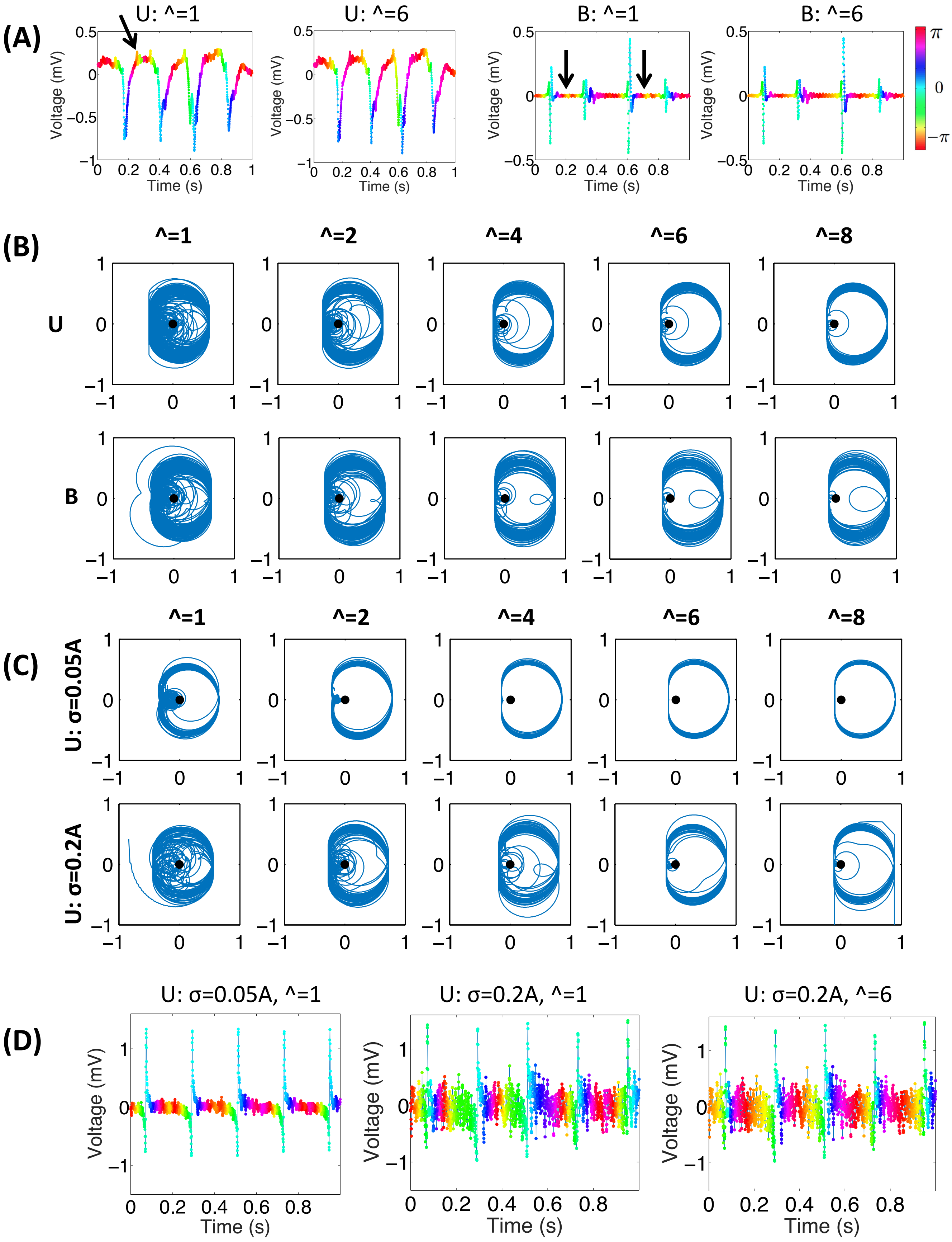
(A)
U: ^=1
U: ^=6
B: ^=1
B: ^=6
Voltage (mV)
Time (s)
π
0
−π
(B)
^=1
^=2
^=4
^=6
^=8
U
B
(C)
U: σ=0.05A
U: σ=0.2A
(D)
U: σ=0.05A, ^=1
U: σ=0.2A, ^=1
U: σ=0.2A, ^=6
Voltage (mV)
Time (s)

S-Fig 2: The effects of exponent of normalisation on phase calculation.
A) For clinical unipolar (left two columns) and bipolar electrograms (right hand columns), phase fluctuations are seen for an exponent of (^) 1 that are not there at 6.
B) Phase loop trajectories for clinical unipolar and bipolar signals, of the zero-mean signal against its Hilbert transform, for increasing exponents.
C) Phase loop trajectories for the zero-mean signal against its Hilbert transform for increasing exponents for a simulated unipolar electrogram with a low level of noise (top) and high level of noise (bottom).
D) Electrogram phase for simulated unipolar electrograms with low noise (left) show some fluctuations for an exponent of 1, which are more pronounced for a high noise level (middle). Increasing the exponent to 6 assigns the phase correctly (right).

Interpolation:

Fig 3 shows the effects of choosing linear or cubic interpolation of phase. There are an increased number of false detections for linear interpolation, motivating the choice of cubic interpolation.

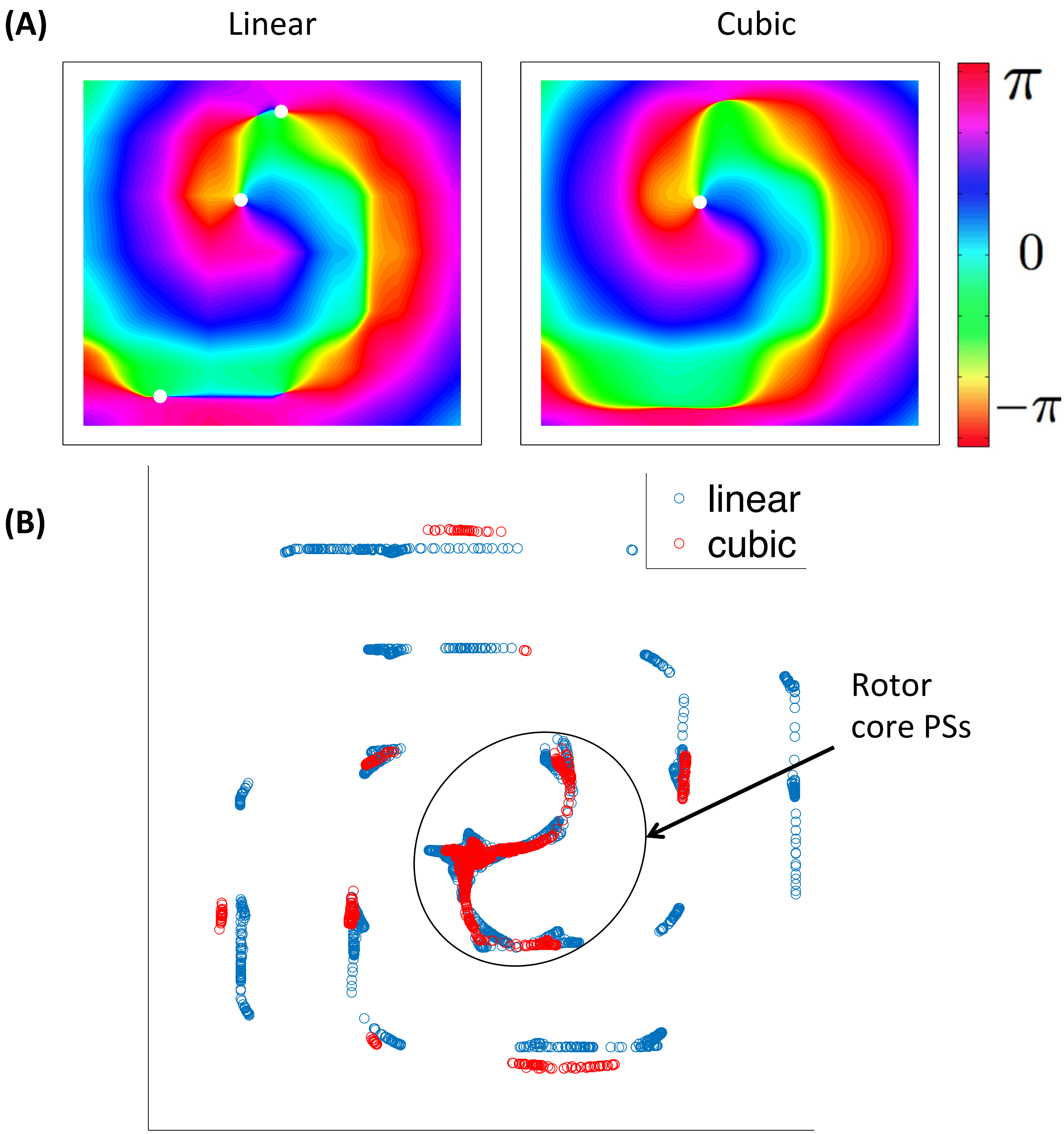

S-Fig 3: Comparison of isophase maps from linear and cubic interpolation techniques: cubic interpolation gives the fewest false detections. *The data were interpolated from 15mm to 0.1mm resolution using linear and cubic interpolation. There are extra phase singularities for the linear case compared to the cubic interpolation. (B) Overall distribution of phase singularities; there are many more false detections for linear interpolation than for cubic interpolation. These false detections tend to occur along the gridlines, outside of the marked rotor-core area.*

Error:

Here we calculate an alternative measure to circular correlation (see “Phase Correlation” section of main manuscript) for calculating the difference between unipolar and bipolar phase recordings. We computed the *error* as the sum over the grid of the difference between the phase images. We also looked at the average difference image over time to see if there were hotspots where the difference was most severe.

For simulated data, the mean difference image between unipolar and bipolar phase over the simulation duration (S-Fig 4A) illustrates there is a high difference close to the average rotor core location. As such, this measure may have applicability in locating stable rotor cores. The overall mean difference between action potential phase and either unipolar or bipolar phase is high (1.65 ± 0.20 rad, 1.66 ± 0.20 rad). This is because it is difficult to identify stages of repolarisation in atrial electrograms [2] and so electrogram phase is not expected to match action potential phase during AF. The mean difference between unipolar and bipolar phase angles is much smaller (0.12 ± 0.06 rad), demonstrating a closer agreement.

For MEA data, the error is seen to vary over time (compare S-Fig 4B to the corresponding plot for circular correlation in the main manuscript, Fig 4A), with a median of 0.16 and a maximum of 0.42, indicating a small average difference in phase values measured with bipolar compared to unipolar recordings.

S-Fig. 4C is a histogram of the number of catheter locations with different error values (compare to Fig 7B, main manuscript). Plotting the error against the circular correlation coefficient shows a linear relationship (S-Fig 4D).

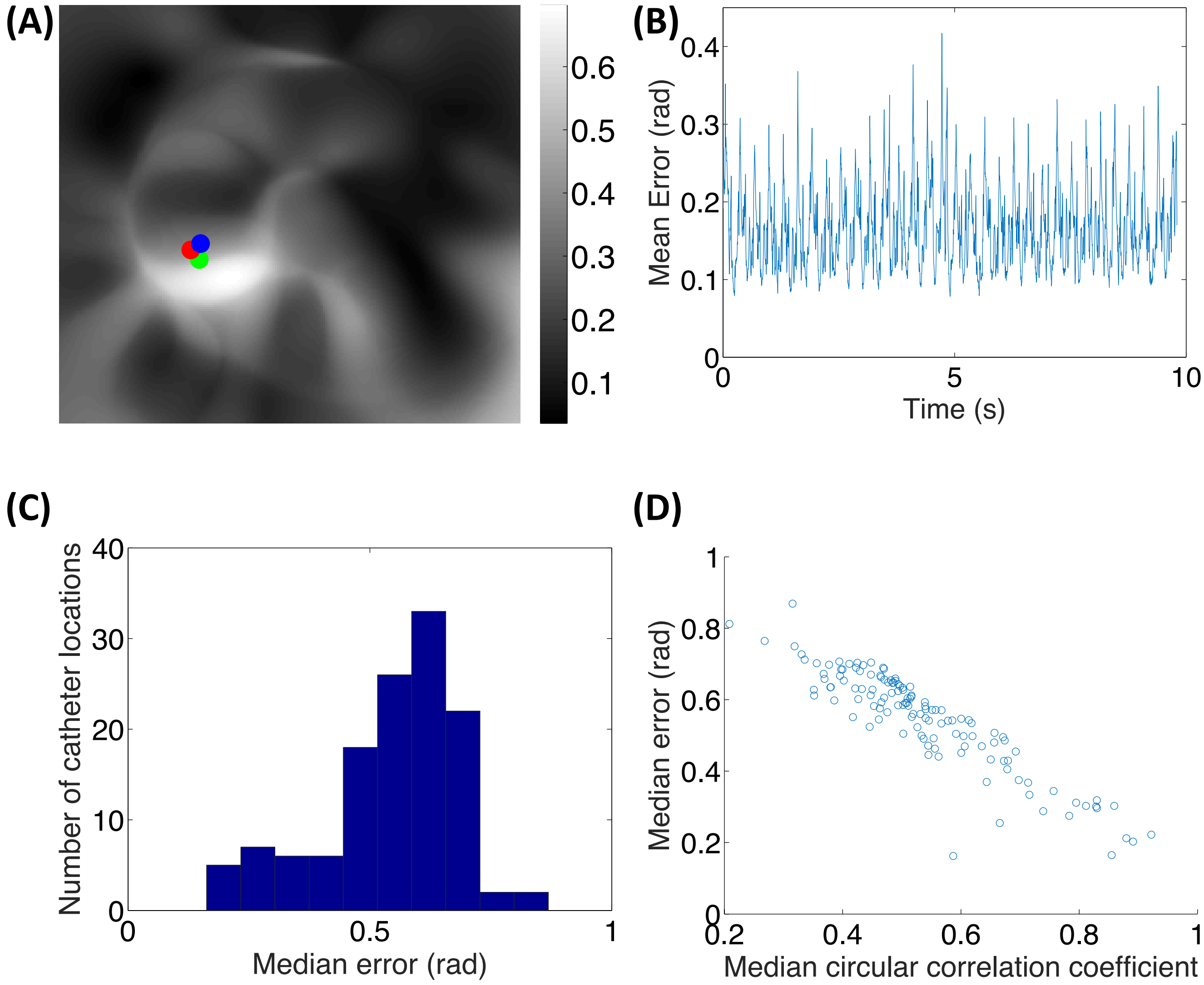

S-Fig 4: Error measured between unipolar and bipolar phase calculated for simulated, MEA and clinical electrograms, for comparison with circular correlation.

A) For the catheter in Fig 3B of the main manuscript, the average difference in unipolar and bipolar phase maps was calculated across the simulation duration. There is an area of high error close to the rotor trajectory centres marked as dots (AP: blue, unipolar: red, bipolar: green).

B) The average error across the electrode array varies over time but is generally low, which agrees with the high correlation seen in Fig 4 of the main manuscript.

C) Histogram to show the distribution of the mean error measured between unipolar and bipolar phase for each of the 127 catheter recordings (compare Fig 7B of main manuscript).

D) A linear relationship is observed between the clinical error and circulation correlation.

References:

[1] M.-A. Bray and J. Wikswo, "Considerations in phase plane analysis for nonstationary reentrant cardiac behavior," *Phys. Rev. E*, vol. 65, no. 5, p. 51902, May 2002.

[2] E. J. Vigmond, V. Tsoi, Y. Yin, P. Pagé, and A. Vinet, "Estimating atrial action potential duration from electrograms.," *IEEE Trans. Biomed. Eng.*, vol. 56, no. 5, pp. 1546–55, May 2009.
